# 3,5-Diamino-1-phenyl-1,2,4-triazolium bromide

**DOI:** 10.1107/S1600536810021318

**Published:** 2010-06-16

**Authors:** V. M. Chernyshev, A. V. Astakhov, V. V. Ivanov, Z. A. Starikova

**Affiliations:** a South-Russia State Technical University, 346428 Novocherkassk, Russian Federation; b A. N. Nesmeyanov Institute of Organoelement Compounds, 119991 Moscow, Russian Federation

## Abstract

The title salt, C_8_H_10_N_5_^+^·Br^−^, crystallizes with two independent structural units in the asymmetric unit. The two independent cations have different conformations, the triazole and phenyl rings forming dihedral angles of 32.57 (6) and 52.27 (7)°. In both cations, the amino groups are planar (the sum of the angles at the N atom of each amino group is 360°) and conjugated with the triazole ring. Inter­molecular N—H⋯N and N—H⋯Br hydrogen bonds consolidate the crystal packing.

## Related literature

For the crystal structures of protonated *C*-amino-1,2,4-triazoles, see: Reck *et al.* (1982 ▶); Lynch *et al.* (1998 ▶, 1999 ▶); Baouab *et al.* (2000 ▶); Bichay *et al.* (2006 ▶); Guerfel *et al.* (2007 ▶); Matulková *et al.* (2007 ▶). For the crystal structure of 3,5-diamino-1,2,4-triazole, see: Starova *et al.* (1980 ▶). For the theoretical investigation of the protonation of *C*-amino-1,2,4-triazoles, see: Anders *et al.* (1997 ▶). For the reactions of 1-substituted 3,5-diamino-1,2,4-triazoles with electrophilic reagents, see: Steck *et al.* (1958 ▶); Chernyshev *et al.* (2005 ▶, 2008 ▶). For the use of 1-substituted 3,5-diamino-1,2,4-triazoles as building blocks in the synthesis of various derivatives of 1,2,4-triazole and fused heterocyclic systems, see: Dunstan *et al.* (1998 ▶); Chernyshev *et al.* (2006 ▶, 2009 ▶, 2010 ▶). For a description of the Cambridge Structural Database, see: Allen (2002 ▶).
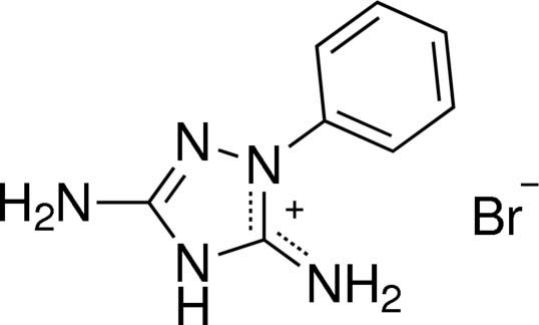


## Experimental

### 

#### Crystal data

C_8_H_10_N_5_^+^·Br^−^
*M* *_r_* = 256.12
Monoclinic, 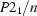

*a* = 13.752 (2) Å
*b* = 7.1172 (13) Å
*c* = 20.394 (4) Å
β = 95.519 (3)°
*V* = 1986.7 (6) Å^3^
*Z* = 8
Mo *K*α radiation
μ = 4.11 mm^−1^
*T* = 100 K
0.55 × 0.40 × 0.30 mm

#### Data collection

Bruker APEXII CCD area-detector diffractometer
Absorption correction: multi-scan (*SADABS*; Bruker, 2004 ▶) *T*_min_ = 0.211, *T*_max_ = 0.372
19484 measured reflections
4314 independent reflections
3808 reflections with *I* > 2σ(*I*)
*R*_int_ = 0.033

#### Refinement

*R*[*F* ^2^ > 2σ(*F* ^2^)] = 0.027
*wR*(*F* ^2^) = 0.071
*S* = 1.00
4314 reflections
253 parameters
H-atom parameters constrained
Δρ_max_ = 0.63 e Å^−3^
Δρ_min_ = −0.52 e Å^−3^

### 

Data collection: *APEX2* (Bruker, 2004 ▶); cell refinement: *SAINT* (Bruker, 2004 ▶); data reduction: *SAINT* and *XPREP* (Bruker, 2005 ▶); program(s) used to solve structure: *SHELXS97* (Sheldrick, 2008 ▶); program(s) used to refine structure: *SHELXL97* (Sheldrick, 2008 ▶); molecular graphics: *Mercury* (Macrae *et al.*, 2006 ▶); software used to prepare material for publication: *SHELXTL* (Sheldrick, 2008 ▶), *publCIF* (Westrip, 2010 ▶) and *PLATON* (Spek, 2009 ▶).

## Supplementary Material

Crystal structure: contains datablocks I, global. DOI: 10.1107/S1600536810021318/cv2726sup1.cif

Structure factors: contains datablocks I. DOI: 10.1107/S1600536810021318/cv2726Isup2.hkl

Additional supplementary materials:  crystallographic information; 3D view; checkCIF report

## Figures and Tables

**Table 1:** Hydrogen-bond geometry (Å, °)

*D*—H⋯*A*	*D*—H	H⋯*A*	*D*⋯*A*	*D*—H⋯*A*
N3—H3*A*⋯N2′^i^	0.86	2.20	3.037 (3)	164
N3—H3*B*⋯Br1	0.86	2.56	3.387 (2)	163
N3′—H3′*A*⋯N2^ii^	0.86	2.34	3.046 (3)	140
N3′—H3′*B*⋯Br2	0.86	2.65	3.404 (3)	147
N4—H4⋯Br2	0.86	2.74	3.417 (3)	137
N4′—H4′⋯Br2	0.86	2.51	3.254 (3)	145
N5—H5*A*⋯Br1^iii^	0.86	2.69	3.369 (3)	137
N5—H5*B*⋯Br2	0.86	2.49	3.281 (3)	153
N5′—H5′*A*⋯Br1^iv^	0.86	2.84	3.489 (3)	133
N5′—H5′*B*⋯Br1	0.86	2.43	3.278 (3)	167

Symmetry codes: (i) 
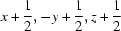
; (ii) 
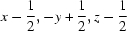
; (iii) 
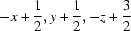
; (iv) 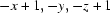.

**Fig. 1.**
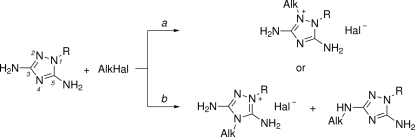
The supposed directions of quaternization of 1-substituted 3,5-diamino-1,2,4-triazoles by halogen alkanes according to the literature data: a - Steck et al. (1958), b - Chernyshev et al. (2008).

**Fig. 2.**
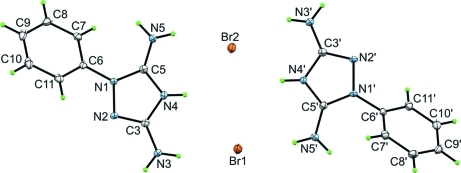
The molecular structure of 1-phenyl-1H-1,2,4-triazole-3,5-diamine hydrobromide with the atomic numbering scheme. Displacement ellipsoids are drawn at the 50% probability level.

**Fig. 3.**
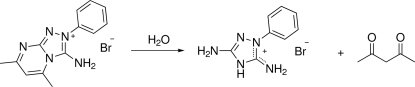
Hydrolysis of 3-amino-5,7-dimethyl-2-phenyl-[1,2,4]triazolo[4,3-a]pyrimidin-2-ium bromide.

**Fig. 4.**
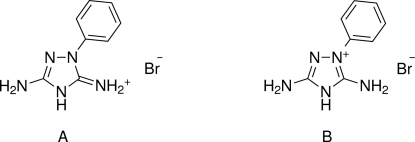
The resonance structures, corresponding to the 5-amino-2-phenyl-2,4-dihydro-3H-1,2,4-triazol-3-iminium bromide (A) and 3,5-diamino-1-phenyl-4H-1,2,4-triazol-1-ium bromide (B).

**Fig. 5.**
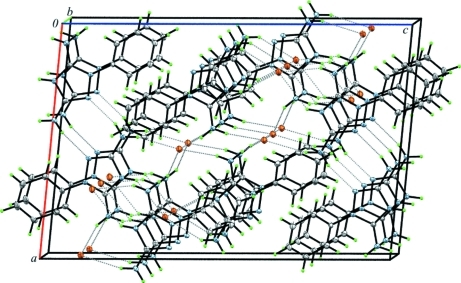
Molecular packing in the crystal, viewed down the b axis. Hydrogen bonds are shown as dashed lines.

**Fig. 6.**
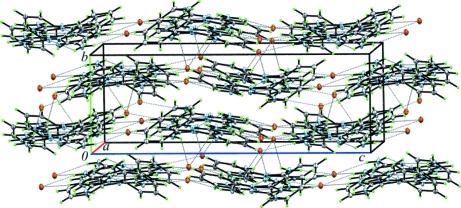
The crystal packing of the title compound, viewed down the a axis showing molecular layers in the planes perpendicular to the b axis. Hydrogen bonds are shown as dashed lines.

## supplementary crystallographic information

### Comment

1-Substituted 3,5-diamino-1,2,4-triazoles are employed as convenient models for investigating the reactions of *C*-amino-1,2,4-triazoles with electrophiles so far as their molecules contain two amino groups having greatly varied nucleophilicity in positions 3 and 5 of triazole cycle (Chernyshev *et al.*, 2005, 2008, 2010). Relatively high reactivity toward electrophiles allows to use 1-substituted 3,5-diamino-1,2,4-triazoles as starting materials for the selective synthesis of 1,2,4-triazole derivatives and annulated heterocycles (Dunstan *et al.*, 1998; Chernyshev *et al.*, 2006, 2009, 2010). Some contradictions concerning the direction of several reactions of these compounds with electrophiles are present in the literature. For example, it was reported that quaternization of 1-substituted 3,5-diamino-1,2,4-triazoles by alkyl halides (Steck *et al.*, 1958) resulted in formation of 1,2-disubstituted 3,5-diamino-1,2,4-triazolium salts (Fig. 1). However these data are in contrast with quantum chemical calculations and synthetic experiments according to which the quaternization of 1-substituted 3-amino-1,2,4-triazoles as well as 1-substituted 5-amino-1,2,4-triazoles occurs at the atom N4 of triazole cycle (Anders *et al.*; 1997). While studying the quaternization of 2-amino-4,5,6,7-tetrahydro-[1,2,4]triazolo[1,5-*a*]pyrimidines (Chernyshev *et al.*, 2008), which are analogous to 1-substituted 3,5-diamino-1,2,4-triazoles in most of reactions with electrophiles, we found that alkylation takes place at the atom N4 of triazole cycle (a prevailing product) and at the C3-amino group (a minor product). The quantum chemical calculations predict that the direction of protonation and quaternization of 1-substituted *C*-amino-1,2,4-triazoles should be identical (Anders *et al.*, 1997). Therefore protonation can be used as model reaction for investigation of quaternization of 1-substituted 3,5-diamino-1,2,4-triazoles by alkyl halides. It should be noted that the data concerning the crystal structure of salts of 1-substituted *C*-amino-1,2,4-triazoles with proton acids were absent in the Cambridge Structural Database so far (Allen, 2002). Here we report the crystal structure of the title compound, 1-phenyl-1*H*-1,2,4-triazole-3,5-diamine hydrobromide (Fig. 2). The crystals of this compound were surprisingly obtained in attempting to grow the crystal of 3-amino-5,7-dimethyl-2-phenyl-[1,2,4]triazolo[4,3-*a*]pyrimidin-2-ium bromide, suitable for X-ray investigation, from water-acetonitrile. Obviously, the starting triazolopyrimidine gradually hydrolyzed to the 1-phenyl-1*H*-1,2,4-triazole-3,5-diamine hydrobromide and 2,4-pentanedion. It indicates that 1-substituted [1,2,4]triazolo[4,3-*a*]pyrimidin-2-ium salts are inclined to hydrolyze even at room temperature (Fig. 3).

According to our X-ray investigation, the asymmetric unit of the crystal structure consists of two crystallographically independent cations further denoted as the cation A (N1N2C3··· *etc*.) and the cation B (N1'N2'C3'··· *etc*.), and two bromide anions (Br1 and Br2). The cations A and B somewhat differ in bond lengths and mutual orientation of benzene and triazole rings. The dihedral angle between the benzene and triazole cycles is 32.57 (6)° in the cation A whereas that is 52.27 (7)° in the cation B. The triazole cycle is planar in both cations (the deviation of atoms from the mean-square planes does not exceed 0.008 (2) Å). As with the other salts of *C*-amino-1,2,4-triazoles (Reck *et al.*, 1982; Lynch *et al.*, 1998, 1999; Baouab *et al.*, 2000; Bichay *et al.*, 2006; Guerfel *et al.*, 2007; Matulková *et al.*, 2007), the acid proton is attached to the atom N4, amino groups are planar and conjugated with the π-system of triazole cycle. In contrast to the unprotonated 3,5-diamino-1,2,4-triazole (Starova *et al.*, 1980) and alkyl derivatives of 3,5-diamino-1-phenyl-1,2,4-triazole (Dunstan *et al.*, 1998), in the cations A and B the C5—N1 and C3—N2 bonds are shorter than the C3—N4 and C5—N4 bonds. An analogous regularity is observed for the majority of other salts of *C*-amino-1,2,4-triazoles (Reck *et al.*, 1982; Lynch *et al.*, 1998, 1999; Baouab *et al.*, 2000; Bichay *et al.*, 2006; Guerfel *et al.*, 2007; Matulková *et al.*, 2007). Thus, the majority of protonated *C*-amino-1,2,4-triazoles should be considered as derivatives of 4*H*-1,2,4-triazol-1-ium rather than 1*H*-1,2,4-triazol-4-ium cation, except of 5-amino-3-azido-1*H*-1,2,4-triazol-4-ium nitrate (Bichay *et al.*, 2006). It is remarkable that the bond C5—N5 (1.325 (3) Å) in the cation A is shorter than the bonds C5—N1 (1.335 (3) Å) and C5—N4 (1.358 (3) Å). The analysis of bond lengths indicates that molecules forming by cation A can be described in the best way by the resonance structure of 5-amino-2-phenyl-2,4-dihydro-3*H*-1,2,4-triazol-3-iminium bromide (Fig. 4). A similar distributions of bond lengths are observed in many other salts of *C*-amino-1,2,4-triazoles (for example, see: Reck *et al.*, 1982; Lynch *et al.*, 1998, 1999; Bichay *et al.*, 2006; Matulková *et al.*, 2007; Guerfel *et al.*, 2007). Therefore, it can be concluded that the C5—NH_2_ group plays an important role in the redistribution of positive charge in the *C*-amino-1,2,4-triazolium cations. Molecules including cation B are properly described by the resonance structure of 3,5-diamino-1-phenyl-4*H*-1,2,4-triazol-1-ium bromide (Fig. 4).

In the crystal the identical and parallel cations of type A or B form stacks along the *b* axis of the monoclinic cell (Fig. 5). In the direction [101] the adjacent stacks of the different-type cations form pairs in which they are displaced from each other on 0.5 cell parameter *b*. One-type cations from the nearest stacks are related in the same direction by a glide plane n perpendicular to [0, 1, 0] with glide component [1/2, 0, 1/2]. In the direction *c* the cations are turned from each other by 180° and displaced on 0.5 of cell parameter, *i.e.* are space related by 2-fold screw axis with direction [0, 1, 0] at 1/4, *y*, 1/4 with screw component [0, 1/2, 0]. Along the *c* axis one can see parallel linear chains which "links" consist of pairs of cations A and B connected with bromide anions Br1 and Br2 by means of the hydrogen bonds N3—H3B···Br1, N5'—H5'B···Br1, N4—H4···Br2, N4'—H4'···Br2, N5—H5B···Br2, N3'—H3'B···Br2 (Table 1). The nearest chains in the plane perpendicular to *b* axis are connected with each other by continuous net of hydrogen bonds N3—H3A···N2'^i^ and N3'—H3'A···N2^ii^, forming parallel molecular layers with identity period equal to the unit-cell parameter *b* (Fig. 6). The layers are connected with one another by hydrogen bonds N5—H5A···Br1^iii^ and N5'—H5'A···Br1^iv^. In the parallel layers one-type cations are turned from each other by 180°, *i.e.* they are space related by inversion centre with coordinates [0, 0, 0]. Thereby, the C_8_H_10_N_5_^+^ cations and bromide anions form a three-dimensional framework in the crystal.

In conclusion, the present study and previously reported theoretical (Anders *et al.*, 1997) and experimental (Chernyshev *et al.*, 2008) results indicate that the structures attributed to the products of quaternization of 1-substituted 3,5-diamino-1,2,4-triazoles (Steck *et al.*, 1958), apparently, are erroneous and need correction by means of modern analytical methods. Also it would be interesting to investigate the structure of salts of another 1-substituted 3-amino-1,2,4-triazoles with a view to evaluate the role of C3—NH_2_ group in the delocalization of positive charge in 3-amino-1,2,4-triazolium cations.

### Experimental

The crystals of 1-phenyl-1*H*-1,2,4-triazole-3,5-diamine hydrobromide suitable for X-ray analysis were obtained from a solution of 3-amino-5,7-dimethyl-2-phenyl-[1,2,4]triazolo[4,3-*a*]pyrimidin-2-ium bromide (TPB) in 1:9 water: acetonitrile mixture as a result of hydrolysis in the course of slow evaporation at room temperature during one week. The TPB was prepared by the following procedure.

A mixture of 1-phenyl-1*H*-1,2,4-triazole-3,5-diamine hydrobromide (0.73 g, 2.85 mmol), 2,4-pentanedion (0.371 g, 3.71 mmol) and ethanol (5 ml) was refluxed for 15 min and then cooled to room temperature. The precipitate formed was filtered off and recrystallized from ethanol to give 0.757 g (83% yield) of TPB, mp 221–223 °C. ^1^H NMR (300 MHz) *δ*: 1.95 (s, 3H, CH_3_), 2.95 (s, 3H, CH_3_), 7.22 (s, 1H, CH), 7.67–7.83 (m, 5H, Ph), 8.37 (s, 2H, NH_2_). ^13^C NMR (150 MHz) *δ*: 17.66, 24.37, 113.62, 130.13, 130.30, 132.16, 132.63, 147.86, 153.85, 159.74, 170.43. LCMS: 240.29 [C_13_H_14_N_5_^+^]. Anal. Calcd for C_13_H_14_BrN_5_: C, 48.76; H, 4.41; N, 21.87. Found: C, 48.81; H, 4.21; N, 21.98.

Starting 1-phenyl-1*H*-1,2,4-triazole-3,5-diamine hydrobromide used for the preparation of TPB was obtained by addition of equimolar amount of 48% hydrobromic acid to an ethanol solution of 3,5-diamino-1-phenyl-1,2,4-triazole. The latter compound was synthesized by known method (Steck *et al.*, 1958).

### Refinement

C-bound H atoms were positioned geometrically (C—H 0.93 Å), while the rest H atoms were located on difference map and further placed in idealized positions (N—H 0.86 Å). All H atoms were refined as riding on their parent atoms, with *U*_iso_(H) = 1.2 *U*_eq_(parent atom).

### Crystal data

**Table:**

C_8_H_10_N_5_^+^·Br^−^	*F*(000) = 1024
*M**_r_* = 256.12	*D*_x_ = 1.713 Mg m^−^^3^
Monoclinic, *P*2_1_/*n*	Mo *K*α radiation, λ = 0.71073 Å
Hall symbol: -P 2yn	Cell parameters from 568 reflections
*a* = 13.752 (2) Å	θ = 3–26°
*b* = 7.1172 (13) Å	µ = 4.11 mm^−^^1^
*c* = 20.394 (4) Å	*T* = 100 K
β = 95.519 (3)°	Plate, colourless
*V* = 1986.7 (6) Å^3^	0.55 × 0.40 × 0.30 mm
*Z* = 8	

### Data collection

**Table:**

Bruker APEXII CCD area-detector diffractometer	4314 independent reflections
Radiation source: fine-focus sealed tube	3808 reflections with *I* > 2σ(*I*)
graphite	*R*_int_ = 0.033
ω scans	θ_max_ = 27.0°, θ_min_ = 1.7°
Absorption correction: multi-scan (*SADABS*; Bruker, 2004)	*h* = −17→17
*T*_min_ = 0.211, *T*_max_ = 0.372	*k* = −9→9
19484 measured reflections	*l* = −26→26

### Refinement

**Table:**

Refinement on *F*^2^	Primary atom site location: structure-invariant direct methods
Least-squares matrix: full	Secondary atom site location: difference Fourier map
*R*[*F*^2^ > 2σ(*F*^2^)] = 0.027	Hydrogen site location: difference Fourier map
*wR*(*F*^2^) = 0.071	H-atom parameters constrained
*S* = 1.00	*w* = 1/[σ^2^(*F*_o_^2^) + (0.0375*P*)^2^ + 2.843*P*] where *P* = (*F*_o_^2^ + 2*F*_c_^2^)/3
4314 reflections	(Δ/σ)_max_ = 0.001
253 parameters	Δρ_max_ = 0.63 e Å^−^^3^
0 restraints	Δρ_min_ = −0.52 e Å^−^^3^

### Special details

**Table:**

Geometry. All e.s.d.'s (except the e.s.d. in the dihedral angle between two l.s. planes) are estimated using the full covariance matrix. The cell e.s.d.'s are taken into account individually in the estimation of e.s.d.'s in distances, angles and torsion angles; correlations between e.s.d.'s in cell parameters are only used when they are defined by crystal symmetry. An approximate (isotropic) treatment of cell e.s.d.'s is used for estimating e.s.d.'s involving l.s. planes.
Refinement. Refinement of *F*^2^ against ALL reflections. The weighted *R*-factor *wR* and goodness of fit *S* are based on *F*^2^, conventional *R*-factors *R* are based on *F*, with *F* set to zero for negative *F*^2^. The threshold expression of *F*^2^ > σ(*F*^2^) is used only for calculating *R*-factors(gt) *etc*. and is not relevant to the choice of reflections for refinement. *R*-factors based on *F*^2^ are statistically about twice as large as those based on *F*, and *R*- factors based on ALL data will be even larger.

### Fractional atomic coordinates and isotropic or equivalent isotropic displacement parameters (Å^2^)

**Table:**

	*x*	*y*	*z*	*U*_iso_*/*U*_eq_	
Br1	0.469797 (17)	0.08357 (3)	0.629576 (11)	0.01647 (7)	
Br2	0.183938 (18)	0.24667 (3)	0.647848 (11)	0.01826 (8)	
N1'	0.28802 (13)	0.3108 (3)	0.40098 (9)	0.0106 (4)	
N1	0.33201 (14)	0.2566 (3)	0.88547 (9)	0.0105 (4)	
C5	0.28387 (16)	0.2595 (3)	0.82544 (11)	0.0109 (4)	
C3'	0.17571 (16)	0.3375 (3)	0.46553 (11)	0.0109 (4)	
N2	0.43255 (13)	0.2227 (3)	0.88259 (9)	0.0110 (4)	
N2'	0.18826 (13)	0.3526 (3)	0.40275 (9)	0.0110 (4)	
C3	0.44019 (16)	0.2009 (3)	0.81952 (11)	0.0112 (4)	
C5'	0.33040 (16)	0.2696 (3)	0.46079 (11)	0.0108 (4)	
N3	0.52349 (14)	0.1671 (3)	0.79256 (10)	0.0155 (4)	
H3A	0.5776	0.1581	0.8172	0.019*	
H3B	0.5228	0.1544	0.7506	0.019*	
C6	0.29678 (17)	0.2856 (3)	0.94788 (11)	0.0112 (4)	
C6'	0.33268 (16)	0.3120 (3)	0.34068 (11)	0.0108 (4)	
N3'	0.09158 (14)	0.3663 (3)	0.49263 (10)	0.0139 (4)	
H3'A	0.0397	0.3978	0.4682	0.017*	
H3'B	0.0897	0.3533	0.5344	0.017*	
C7'	0.41759 (17)	0.4159 (3)	0.33736 (12)	0.0141 (4)	
H7'	0.4425	0.4895	0.3727	0.017*	
N4'	0.26139 (14)	0.2888 (3)	0.50273 (9)	0.0106 (4)	
H4'	0.2691	0.2737	0.5447	0.013*	
N4	0.35127 (14)	0.2222 (3)	0.78272 (9)	0.0110 (4)	
H4	0.3406	0.2134	0.7406	0.013*	
C7	0.20341 (17)	0.2241 (3)	0.95945 (12)	0.0144 (5)	
H7	0.1630	0.1663	0.9262	0.017*	
C8'	0.46478 (17)	0.4074 (3)	0.27991 (12)	0.0168 (5)	
H8'	0.5225	0.4738	0.2771	0.020*	
N5	0.18967 (14)	0.2885 (3)	0.80841 (10)	0.0139 (4)	
H5A	0.1508	0.3091	0.8382	0.017*	
H5B	0.1674	0.2869	0.7675	0.017*	
N5'	0.42386 (14)	0.2226 (3)	0.47651 (10)	0.0140 (4)	
H5'A	0.4634	0.2166	0.4464	0.017*	
H5'B	0.4445	0.1983	0.5168	0.017*	
C8	0.17218 (18)	0.2512 (4)	1.02182 (12)	0.0189 (5)	
H8	0.1098	0.2134	1.0300	0.023*	
C9'	0.42605 (18)	0.3005 (4)	0.22696 (12)	0.0184 (5)	
H9'	0.4585	0.2932	0.1891	0.022*	
C9	0.2331 (2)	0.3340 (4)	1.07174 (12)	0.0203 (5)	
H9	0.2116	0.3514	1.1132	0.024*	
C10'	0.33847 (19)	0.2038 (3)	0.23038 (12)	0.0177 (5)	
H10'	0.3116	0.1362	0.1940	0.021*	
C10	0.32641 (19)	0.3909 (3)	1.05977 (12)	0.0184 (5)	
H10	0.3677	0.4445	1.0935	0.022*	
C11	0.35831 (17)	0.3681 (3)	0.99777 (11)	0.0142 (5)	
H11	0.4205	0.4078	0.9897	0.017*	
C11'	0.29117 (17)	0.2074 (3)	0.28729 (11)	0.0135 (4)	
H11'	0.2332	0.1418	0.2899	0.016*	

### Atomic displacement parameters (Å^2^)

**Table:**

	*U*^11^	*U*^22^	*U*^33^	*U*^12^	*U*^13^	*U*^23^
Br1	0.01631 (12)	0.02134 (13)	0.01186 (12)	−0.00248 (9)	0.00199 (8)	0.00081 (9)
Br2	0.02081 (13)	0.02303 (14)	0.01122 (12)	0.00564 (10)	0.00289 (9)	−0.00001 (9)
N1'	0.0066 (9)	0.0153 (9)	0.0099 (9)	−0.0003 (7)	0.0012 (7)	0.0007 (7)
N1	0.0081 (9)	0.0145 (9)	0.0089 (9)	0.0022 (7)	0.0008 (7)	−0.0006 (7)
C5	0.0112 (10)	0.0094 (10)	0.0122 (10)	−0.0009 (8)	0.0013 (8)	−0.0006 (8)
C3'	0.0107 (10)	0.0097 (10)	0.0123 (10)	−0.0016 (8)	0.0008 (8)	−0.0007 (8)
N2	0.0074 (9)	0.0150 (9)	0.0108 (9)	0.0020 (7)	0.0020 (7)	−0.0005 (7)
N2'	0.0064 (9)	0.0146 (9)	0.0122 (9)	0.0006 (7)	0.0016 (7)	0.0012 (7)
C3	0.0129 (11)	0.0092 (10)	0.0115 (10)	0.0011 (8)	0.0014 (8)	0.0000 (8)
C5'	0.0110 (10)	0.0106 (10)	0.0109 (10)	−0.0014 (8)	0.0017 (8)	−0.0001 (8)
N3	0.0117 (9)	0.0250 (11)	0.0100 (9)	0.0050 (8)	0.0028 (7)	−0.0012 (8)
C6	0.0143 (11)	0.0106 (10)	0.0090 (10)	0.0053 (8)	0.0027 (8)	0.0015 (8)
C6'	0.0117 (10)	0.0117 (10)	0.0092 (10)	0.0030 (8)	0.0023 (8)	0.0026 (8)
N3'	0.0105 (9)	0.0209 (10)	0.0106 (9)	0.0019 (8)	0.0027 (7)	−0.0005 (8)
C7'	0.0134 (11)	0.0144 (11)	0.0146 (11)	0.0005 (9)	0.0014 (8)	0.0017 (9)
N4'	0.0111 (9)	0.0139 (9)	0.0069 (9)	−0.0009 (7)	0.0012 (7)	−0.0004 (7)
N4	0.0114 (9)	0.0141 (9)	0.0076 (9)	0.0014 (7)	0.0008 (7)	−0.0005 (7)
C7	0.0120 (11)	0.0169 (11)	0.0141 (11)	0.0040 (9)	0.0008 (9)	0.0038 (9)
C8'	0.0119 (11)	0.0193 (12)	0.0197 (12)	0.0013 (9)	0.0045 (9)	0.0094 (10)
N5	0.0102 (9)	0.0214 (10)	0.0097 (9)	0.0024 (8)	−0.0009 (7)	−0.0026 (8)
N5'	0.0099 (9)	0.0212 (10)	0.0108 (9)	0.0015 (8)	−0.0002 (7)	0.0021 (8)
C8	0.0160 (12)	0.0233 (13)	0.0187 (12)	0.0094 (10)	0.0079 (9)	0.0094 (10)
C9'	0.0202 (12)	0.0225 (12)	0.0137 (11)	0.0086 (10)	0.0084 (9)	0.0073 (10)
C9	0.0297 (14)	0.0207 (12)	0.0112 (11)	0.0139 (11)	0.0065 (10)	0.0030 (9)
C10'	0.0222 (12)	0.0182 (12)	0.0126 (11)	0.0070 (10)	0.0004 (9)	−0.0006 (9)
C10	0.0268 (13)	0.0161 (12)	0.0118 (11)	0.0074 (10)	−0.0010 (9)	−0.0016 (9)
C11	0.0158 (11)	0.0121 (10)	0.0143 (11)	0.0039 (9)	−0.0003 (9)	0.0000 (9)
C11'	0.0138 (11)	0.0137 (11)	0.0130 (11)	0.0009 (9)	0.0002 (9)	0.0011 (9)

### Geometric parameters (Å, °)

**Table:**

N1'—C5'	1.333 (3)	C7'—C8'	1.394 (3)
N1'—N2'	1.408 (2)	C7'—H7'	0.9300
N1'—C6'	1.426 (3)	N4'—H4'	0.8600
N1—C5	1.335 (3)	N4—H4	0.8600
N1—N2	1.410 (3)	C7—C8	1.395 (3)
N1—C6	1.420 (3)	C7—H7	0.9300
C5—N5	1.325 (3)	C8'—C9'	1.385 (4)
C5—N4	1.358 (3)	C8'—H8'	0.9300
C3'—N2'	1.313 (3)	N5—H5A	0.8600
C3'—N3'	1.345 (3)	N5—H5B	0.8600
C3'—N4'	1.383 (3)	N5'—H5'A	0.8600
N2—C3	1.310 (3)	N5'—H5'B	0.8600
C3—N3	1.339 (3)	C8—C9	1.386 (4)
C3—N4	1.380 (3)	C8—H8	0.9300
C5'—N5'	1.337 (3)	C9'—C10'	1.395 (4)
C5'—N4'	1.344 (3)	C9'—H9'	0.9300
N3—H3A	0.8600	C9—C10	1.389 (4)
N3—H3B	0.8600	C9—H9	0.9300
C6—C11	1.389 (3)	C10'—C11'	1.384 (3)
C6—C7	1.398 (3)	C10'—H10'	0.9300
C6'—C7'	1.389 (3)	C10—C11	1.387 (3)
C6'—C11'	1.395 (3)	C10—H10	0.9300
N3'—H3'A	0.8600	C11—H11	0.9300
N3'—H3'B	0.8600	C11'—H11'	0.9300
			
C5'—N1'—N2'	111.37 (18)	C3'—N4'—H4'	126.5
C5'—N1'—C6'	127.14 (19)	C5—N4—C3	107.23 (18)
N2'—N1'—C6'	121.48 (17)	C5—N4—H4	126.4
C5—N1—N2	111.48 (18)	C3—N4—H4	126.4
C5—N1—C6	129.6 (2)	C8—C7—C6	118.6 (2)
N2—N1—C6	118.86 (18)	C8—C7—H7	120.7
N5—C5—N1	129.0 (2)	C6—C7—H7	120.7
N5—C5—N4	125.0 (2)	C9'—C8'—C7'	120.2 (2)
N1—C5—N4	106.04 (19)	C9'—C8'—H8'	119.9
N2'—C3'—N3'	126.0 (2)	C7'—C8'—H8'	119.9
N2'—C3'—N4'	111.76 (19)	C5—N5—H5A	120.0
N3'—C3'—N4'	122.3 (2)	C5—N5—H5B	120.0
C3—N2—N1	103.46 (17)	H5A—N5—H5B	120.0
C3'—N2'—N1'	103.15 (17)	C5'—N5'—H5'A	120.0
N2—C3—N3	125.2 (2)	C5'—N5'—H5'B	120.0
N2—C3—N4	111.75 (19)	H5'A—N5'—H5'B	120.0
N3—C3—N4	123.0 (2)	C9—C8—C7	120.8 (2)
N1'—C5'—N5'	126.9 (2)	C9—C8—H8	119.6
N1'—C5'—N4'	106.66 (19)	C7—C8—H8	119.6
N5'—C5'—N4'	126.5 (2)	C8'—C9'—C10'	120.1 (2)
C3—N3—H3A	120.0	C8'—C9'—H9'	120.0
C3—N3—H3B	120.0	C10'—C9'—H9'	120.0
H3A—N3—H3B	120.0	C8—C9—C10	119.9 (2)
C11—C6—C7	120.9 (2)	C8—C9—H9	120.1
C11—C6—N1	118.8 (2)	C10—C9—H9	120.1
C7—C6—N1	120.3 (2)	C11'—C10'—C9'	120.7 (2)
C7'—C6'—C11'	121.9 (2)	C11'—C10'—H10'	119.7
C7'—C6'—N1'	118.6 (2)	C9'—C10'—H10'	119.7
C11'—C6'—N1'	119.5 (2)	C11—C10—C9	120.3 (2)
C3'—N3'—H3'A	120.0	C11—C10—H10	119.8
C3'—N3'—H3'B	120.0	C9—C10—H10	119.8
H3'A—N3'—H3'B	120.0	C10—C11—C6	119.6 (2)
C6'—C7'—C8'	118.7 (2)	C10—C11—H11	120.2
C6'—C7'—H7'	120.7	C6—C11—H11	120.2
C8'—C7'—H7'	120.7	C10'—C11'—C6'	118.3 (2)
C5'—N4'—C3'	107.03 (18)	C10'—C11'—H11'	120.8
C5'—N4'—H4'	126.5	C6'—C11'—H11'	120.8
			
N2—N1—C5—N5	−179.4 (2)	C11'—C6'—C7'—C8'	−3.1 (3)
C6—N1—C5—N5	−0.8 (4)	N1'—C6'—C7'—C8'	175.9 (2)
N2—N1—C5—N4	1.8 (2)	N1'—C5'—N4'—C3'	1.7 (2)
C6—N1—C5—N4	−179.6 (2)	N5'—C5'—N4'—C3'	−179.7 (2)
C5—N1—N2—C3	−1.7 (2)	N2'—C3'—N4'—C5'	−1.1 (3)
C6—N1—N2—C3	179.49 (19)	N3'—C3'—N4'—C5'	179.6 (2)
N3'—C3'—N2'—N1'	179.3 (2)	N5—C5—N4—C3	−180.0 (2)
N4'—C3'—N2'—N1'	0.0 (2)	N1—C5—N4—C3	−1.1 (2)
C5'—N1'—N2'—C3'	1.2 (2)	N2—C3—N4—C5	0.0 (3)
C6'—N1'—N2'—C3'	−179.8 (2)	N3—C3—N4—C5	−178.4 (2)
N1—N2—C3—N3	179.3 (2)	C11—C6—C7—C8	1.5 (3)
N1—N2—C3—N4	1.0 (2)	N1—C6—C7—C8	178.6 (2)
N2'—N1'—C5'—N5'	179.6 (2)	C6'—C7'—C8'—C9'	1.4 (3)
C6'—N1'—C5'—N5'	0.7 (4)	C6—C7—C8—C9	−1.3 (3)
N2'—N1'—C5'—N4'	−1.8 (2)	C7'—C8'—C9'—C10'	1.3 (4)
C6'—N1'—C5'—N4'	179.2 (2)	C7—C8—C9—C10	0.0 (4)
C5—N1—C6—C11	−147.8 (2)	C8'—C9'—C10'—C11'	−2.4 (4)
N2—N1—C6—C11	30.7 (3)	C8—C9—C10—C11	1.1 (4)
C5—N1—C6—C7	35.1 (3)	C9—C10—C11—C6	−0.9 (3)
N2—N1—C6—C7	−146.4 (2)	C7—C6—C11—C10	−0.4 (3)
C5'—N1'—C6'—C7'	−52.8 (3)	N1—C6—C11—C10	−177.5 (2)
N2'—N1'—C6'—C7'	128.4 (2)	C9'—C10'—C11'—C6'	0.8 (3)
C5'—N1'—C6'—C11'	126.2 (2)	C7'—C6'—C11'—C10'	2.0 (3)
N2'—N1'—C6'—C11'	−52.6 (3)	N1'—C6'—C11'—C10'	−177.0 (2)

### Hydrogen-bond geometry (Å, °)

**Table:**

*D*—H···*A*	*D*—H	H···*A*	*D*···*A*	*D*—H···*A*
N3—H3A···N2'^i^	0.86	2.20	3.037 (3)	164
N3—H3B···Br1	0.86	2.56	3.387 (2)	163
N3'—H3'A···N2^ii^	0.86	2.34	3.046 (3)	140
N3'—H3'B···Br2	0.86	2.65	3.404 (3)	147
N4—H4···Br2	0.86	2.74	3.417 (3)	137
N4'—H4'···Br2	0.86	2.51	3.254 (3)	145
N5—H5A···Br1^iii^	0.86	2.69	3.369 (3)	137
N5—H5B···Br2	0.86	2.49	3.281 (3)	153
N5'—H5'A···Br1^iv^	0.86	2.84	3.489 (3)	133
N5'—H5'B···Br1	0.86	2.43	3.278 (3)	167

Symmetry codes: (i) *x*+1/2, −*y*+1/2, *z*+1/2;  (ii) *x*−1/2, −*y*+1/2, *z*−1/2; (iii) −*x*+1/2, *y*+1/2, −*z*+3/2; (iv) −*x*+1, −*y*, −*z*+1.
